# Birth Weight, Working Memory and Epigenetic Signatures in *IGF2* and Related Genes: A MZ Twin Study

**DOI:** 10.1371/journal.pone.0103639

**Published:** 2014-08-29

**Authors:** Aldo Córdova-Palomera, Silvia Alemany, Mar Fatjó-Vilas, Ximena Goldberg, Juan Carlos Leza, Ana González-Pinto, Igor Nenadic, Lourdes Fañanás

**Affiliations:** 1 Unitat d'Antropologia, Departament de Biologia Animal, Facultat de Biologia and Institut de Biomedicina (IBUB), Universitat de Barcelona, Barcelona, Spain; 2 Centro de Investigaciones Biomédicas en Red de Salud Mental (CIBERSAM), Instituto de Salud Carlos III, Madrid, Spain; 3 Department of Pharmacology, Faculty of Medicine, Universidad Complutense, Madrid, Spain, and Instituto de Investigación Hospital 12 de Octubre (I+12), Madrid, Spain; 4 Psychiatry Service, Santiago Apóstol Hospital, EMBREC, EHU/UPV University of the Basque Country, Vitoria, Spain; 5 Department of Psychiatry and Psychotherapy, Jena University Hospital, Jena, Germany; Harbin Medical University, China

## Abstract

Neurodevelopmental disruptions caused by obstetric complications play a role in the etiology of several phenotypes associated with neuropsychiatric diseases and cognitive dysfunctions. Importantly, it has been noticed that epigenetic processes occurring early in life may mediate these associations. Here, DNA methylation signatures at *IGF2* (insulin-like growth factor 2) and *IGF2BP1-3* (IGF2-binding proteins 1-3) were examined in a sample consisting of 34 adult monozygotic (MZ) twins informative for obstetric complications and cognitive performance. Multivariate linear regression analysis of twin data was implemented to test for associations between methylation levels and both birth weight (BW) and adult working memory (WM) performance. Familial and unique environmental factors underlying these potential relationships were evaluated. A link was detected between DNA methylation levels of two CpG sites in the *IGF2BP1* gene and both BW and adult WM performance. The BW-*IGF2BP1* methylation association seemed due to non-shared environmental factors influencing BW, whereas the WM-*IGF2BP1* methylation relationship seemed mediated by both genes and environment. Our data is in agreement with previous evidence indicating that DNA methylation status may be related to prenatal stress and later neurocognitive phenotypes. While former reports independently detected associations between DNA methylation and either BW or WM, current results suggest that these relationships are not confounded by each other.

## Introduction

Prenatal growth in humans has been linked to several complex disorders in adulthood. In this regard, epidemiological studies demonstrate that poor intrauterine environment induces offspring phenotypes which are characterized by an increased risk of developing different chronic diseases [1]. In particular, indirect markers of prenatal suffering such as low birth weight (BW) have been shown to influence risk for neurodevelopmental disorders involving high cognitive dysfunction, such as schizophrenia and autism [2]–[4]. However, further studies indicate that low BW may not influence risk for other mental conditions such as anxiety or depression [5], [6], probably suggesting some specificity between this risk factor and a number of neurodevelopment-related psychiatric and cognitive outcomes.

Remarkably, recent research has shown that epigenetic processes may mediate associations between environmental insults originating low BW, and several pathological conditions across the human lifespan [7]. Of note, among the different epigenetic marks, DNA methylation is particularly interesting in this context, since there is evidence that large inter-individual differences in methylation levels occur at regions covering mammalian developmental genes, and that this variability may correlate with phenotypic plasticity in changing environments [8]. Importantly, additional investigation on this topic has led to propose that some of these so-called variably methylated regions show temporal stability over periods of years and covary with individual traits [9], probably underlying the relationship between prenatal events and DNA methylation in adulthood [10].

In view of it, studies of the insulin-like growth factors and related genes are relevant as regards neurodevelopmental alterations, as it is widely recognized that the proteins they codify participate in complex signaling pathways affecting fetal and postnatal development [11]. Accordingly, epigenetics research indicates that DNA methylation levels of the insulin-like growth factor 2 (*IGF2*) and related developmental genes are linked to human prenatal insults such as maternal malnutrition and stress, fetal insults and low BW [12]–[16], and correlate with particular neuroanatomical features such as cerebral and cerebellar weight [17]–[19]. From these studies, it is feasible inferring that *IGF2* DNA methylation marks (established early in life and measured in adulthood) influencing fetal growth and development could also have some relationship with adult brain outcomes such as neurocognitive and neuropsychiatric traits.

Accordingly, expression levels, polymorphic variants and other biologically relevant features of the *IGF2* gene have frequently been associated with neurodevelopmentally induced behavioral traits, neurogenesis and cognitive phenotypes [20]–[24]. Of note, among several neurocognitive functions, *IGF2* has repeatedly been linked not only to modulation of memory consolidation and enhancement [25], [26], but also to working memory (WM) performance [21], [24]. WM designates a mechanism by which things are kept in mind when complex tasks are executed [27], and involves the activation of several brain regions implicated in other types of memory [28]. Its basic structure is thought to develop from around 6 years of age through adolescence [29], [30], and it may probably be modified later in response to training [31].

Remarkably, these two previously proposed links are not definitely clear: on one side, locus-specific *IGF2* DNA methylation has been suggested to remain as a fingerprint reflecting fetal growth disturbances; in contrast, though WM performance evolves during later ontogenetic stages, it has also been related to *IGF2* signaling networks. Furthermore, while some studies suggest adverse prenatal events may modify adult WM performance [32], [33] the evidence for a link between BW and WM in the adult general population is still not conclusive [34]. With this background, it is feasible hypothesizing that plasticity of cognitive functioning could somehow arise in response to biochemical alterations left printed early in life as DNA methylation marks.

Notably, published research reports showing relationships between adult *IGF2* methylation and previous fetal development do not typically control for the putative relationship between this adult epigenetic mark and neuropsychological performance, as reflected in psychometric measures. In addition, to the knowledge of authors, studies relating psychometric outcomes and *IGF2* DNA methylation (rather than expression levels or polymorphic variants) are scarce.

Thus, by exploring DNA methylation levels at *IGF2* and in three genes codifying for allied factors (IGF2-binding proteins 1–3, *IGF2BP1-3*), the current study was aimed at evaluating epigenetic correlates of BW and adult WM performance in a monozygotic (MZ) twin sample. Models implemented here assessed a putative link between DNA methylation and either BW or WM, controlling for each other. In addition to testing for direct associations, using MZ twins also allowed evaluating methylation changes and their putative phenotypic correlates controlling for confounding factors common to both twins (i.e. genes and shared environment).

## Materials and Methods

### a. Ethics statement

Written informed consent was obtained from all participants after a detailed description of the study aims and design, approved by the institutional ethics committee (Comissió de Bioètica de la Universitat de Barcelona (CBUB); Institutional Review Board registry IRB00003099; Assurance number: FWA00004225; http://www.ub.edu/recerca/comissiobioetica.htm). All procedures were in accordance with the Declaration of Helsinki.

### b. Sample description

Participants of this study were part of a larger twin sample consisting of 242 European descent Spanish adult twins from the general population who gave permission to be contacted for research purposes. The current sample consisted of a 34-individual (17-twin-pair) subset of participants extracted from the initial group of participants. For the current sample, exclusion criteria applied included age under 21 and over 65, a medical history of neurological disturbance, presence of sensory or motor alterations and current substance misuse or dependence.

Medical records and a battery of psychological and neurocognitive tests were obtained in face-to-face interviews by trained psychologists (S.A and X.G.). Additionally, peripheral blood or saliva samples were obtained from all participants, and zygosity of the pairs was determined by genotyping 16 highly polymorphic microsatellite loci from DNA samples (SSRs; PowerPlex 16 System Promega Corporation). Identity on all the markers can be used to assign monozygosity (i.e., whether twins of a given pair were born from a single fertilized ovum, and are so identical at the DNA sequence level) with greater than 99% accuracy [35].

From the previous sample, a group of 34 middle-aged participants (17 MZ twin pairs; age range 22–56, median age 38; 47% female), who were informative for psychopathology, neurocognition and early stress factors, accepted to participate in an ongoing research project relating cognitive performance, brain function and genome-wide epigenetic signatures. Peripheral blood was available for all members of this group. All analyses described below refer to this 34-individual subset (Table 1).

**Table 1:** Descriptive data for variables included in the analyses.

Total sample		
*n* = 34 (17 MZ twin pairs, 47% female)		
	Mean (SD)	Range
***Individual-level description***		
**Age (years)**	37.8 (11.2)	22–56
**Weeks of gestation**	36.9 (2.4)	30–39
**BW (kilograms)**	2.4 (0.5)	1.4–3.4
**WM score**	110.4 (13.4)	89–142
Methylation fraction*	43.4 (11.9)%	20.7–60.8%
***MZ twin intrapair differences***		
**BW differences (kilograms)**	0.3 (0.3)	0–1
**WM score differences**	8.8 (6.1)	0–17
Methylation fraction* differences	4.4 (6.7)%	0.3–28.8%

MZ  =  monozygotic; SD  =  standard deviation; BW  =  birth weight; WM  =  working memory; IQ  =  intellectual quotient; *: average methylation fraction of cg07075026 and cg20966754 (*IGF2BP1*) (see Materials and methods: b. CpG region selection).

### c. Methylation data

The Illumina Infinium HumanMethylation450 (450K) BeadChip [36], [37] was used. Briefly, by genotyping sodium bisulfite treated DNA, this platform assays DNA methylation at 482,421 CpG sites across the genome at single base resolution; afterwards, bisulfite-converted DNA undergoes whole-genome amplification, before being fragmented and hybridized to microarray probes. Indexes of DNA methylation fraction of each CpG site are estimated as 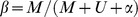; 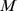 and 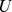 stand for methylated and unmethylated fluorescence intensities, and 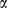 is an arbitrary offset applied to stabilize 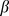 values with low intensities.

### d. CpG region selection

The microarray data contained methylation levels of 248 CpG sites mapped to locations at the four genes of interest (*IGF2* (11p15.5), *IGF2BP1* (17q21.32), *IGF2BP2* (3q27.2), and *IGF2BP3* (7p15.3)) in the human genome (hg19).

High intrapair correlation coefficients in methylation fractions were observed among MZ twin pairs when comparing their 248 CpG sites of interest (Spearman's rho for each of the 17 pairs ranging from 0.973 to 0.993).

Afterwards, variation across each of the 248 regions was evaluated, both at the whole-sample level and considering intrapair differences, in order to define regions with substantial inter-individual variation (i.e., informative variably methylated regions). Briefly, on the basis of a previously described procedure [9], the median absolute deviation (MAD) was estimated for each CpG site considering all 34 individuals. MAD provides a measure of variability in a distribution which is less biased by outliers than standard deviation. In the same way, after calculating the absolute value of the intrapair difference across the 248 CpG sites for the 17 twin pairs, median values (of the differences) were computed. Large median values would indicate the presence of relatively large MZ twin differences at a given CpG, and allow evaluating whether or not inter-individual variation (i.e., in the whole sample) is accompanied by intrapair differences.

**Figure 1:**
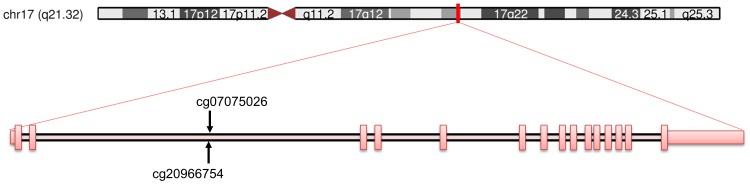
Schematic diagram of the studied CpG sites in the *IGF2BP1* gene. Top: depiction of chromosome 17 with a red mark indicating the locus of *IGF2BP1* (chr17:47,074,774–47,133,012). Bottom: location of cg07075026 and cg20966754 CpG sites, represented over an intron in a transcript of *IGF2BP1* at chr17:47,074,774–47,133,507. Red vertical squares crossing the transcript cover exonic regions. Adapted from the UCSC Genome Browser (GRCh37/hg19; http://genome.ucsc.edu).

Further information about these CpG sites in relation to the UCSC Genome Browser (GRCh37/hg19) [38] coordinates and CpG islands can be found in Fig. 1 and Tables 1 and 2.

**Table 2:** Information of the studied CpG probes.

IlmnID	Chr	GRCh37 coor-dinates	Gene Name (UCSC)	Gene region feature category (UCSC)	CpG island \name (UCSC)	Relation to UCSC CpG Island
cg07075026	17	47091521	*IGF2BP1*	Body	chr17:47091037	Island
					-47091567	
cg20966754	17	47091339	*IGF2BP1*	Body	chr17:47091037	Island
					-47091567	

IlmnID  =  Unique CpG locus identifier from the Illumina CG database.

### e. Obstetric data

Information about obstetric complications was collected by direct interviews with the participants' mothers by means of the Lewis-Murray Obstetric Complications Scale [39]. Long-term maternal recall of obstetric complications has been shown to be accurate enough for the current purposes [40]. From this questionnaire, a continuous measure of BW was obtained, and it was subsequently used along with adjustments for weeks of gestational age, as it may confound statistical associations between DNA methylation and other measures of fetal growth [12]. Also, previous systematic evidence review has pointed gestational age adjustment as an indicator of study quality when assessing relationships between BW and adult outcomes [6]. Use of weeks of gestation in linear regression analysis (see below g. Statistical analyses) is justified in this context since prior research has shown that, within the common gestational age range, fetal growth may be almost linearly related to gestational age [41]. Mean (SD) BW of the 34 individuals was 2.448 kilograms (SD = 0.492 kilograms). Mean intrapair difference of BW was 0.288 kilograms, ranging from 0 to 1 kilogram (Table 1). BW distribution by gestational age of all subjects in the sample was in accordance to a previous report of European descent twins [42].

### f. Neurocognitive assessment

WM performance was estimated from two subtests (digit span and letter number sequencing) of the Wechsler Adult Intelligence Scale (WAIS-III) [43], [44]. As it has been suggested that intelligence quotient (IQ) could be associated with WM [45], [46], it was estimated from five WAIS-III subtests (block design, digit span, matrix reasoning, information and vocabulary), and included as covariate (Table 1).

### g. Statistical analyses

Two different multivariate linear regression tests were performed, using only one methylation fraction outcome selected from the initial pool 248 CpG sites in four candidate genes (see b. CpG region selection and Results). First, considering each individual separately (i.e., correcting for clustered responses from twin families), the association between methylation fraction at a given CpG site (as defined in b. CpG region selection) and both BW and WM. Secondly, unique environmental influences (as derived from MZ twin pair differences) on both BW and WM were studied in relation to methylation fraction, using a regression procedure described elsewhere [47].

Briefly, the regression 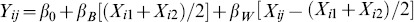 allows estimating both a) familial factors (genes plus shared environment, 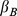) and b) unique environmental influences (non-shared events within a pair, 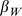) underlying statistical relationships. Subindex 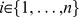 stands for pair number (here, *n* = 17 MZ pairs) and 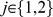 refers to co-twin number (randomly assigned). 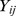 represents the DNA methylation fraction at a given genomic region of co-twin *j* from the *i*-th pair. 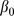 stands for intercept, 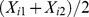 represents the mean BW or WM score of the *i*-th pair and 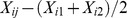 denotes the deviation of co-twin *j* from the pair's mean score.

Gender, age, IQ and weeks of gestation were included as covariates in all analyses. All analyses were performed with R [48]. Linear mixed-effects regressions were executed with package lme4 [49], [50], including family membership as a random effect. Additionally, to reduce the number of regressors, BW and WM were internally adjusted by weeks of gestation and IQ, and all tests were repeated. Since significance of results did not change when introducing this modification, only outcomes from the first set of regressions were considered. Also, as some of the participants showed liability to anxious-depressive psychopathology, analyses were repeated accounting for this fact, but significance of outcomes remained unchanged. Hence, only the former results are presented.

## Results

Variability of methylation fraction across participants at all 248 CpG sites was assessed as both inter-individual (median absolute deviation, MAD) and within-pair (median value of absolute intrapair pair differences) dispersion levels. All 248 sites displayed low intrapair variability (maximum median intrapair difference at a given CpG <|5.5|%). CpG site cg07075026 (in *IGF2BP1*) showed substantially higher inter-individual variability than the other 247 regions (MAD = 0.119); the CpG site with the second largest inter-individual dispersion score (cg20966754, MAD = 0.085) was located in a CpG island (the same as cg07075026), within an intronic region in the gene body of *IGF2BP1* (chr17:47,091,037-47,091,567, see Fig. 1). Consequently, as expected from the physical proximity of these CpG sites, they showed highly correlated values at the intra-individual level (Spearman's rho = 0.956). Thus, a mean methylation value of both sites was used as outcome of interest in all successive calculations. Across the 34 participants, mean methylation fraction for these combined score was 0.43 (SD: 0.12, range: 0.21–0.61).

Although median values of intrapair differences at either cg07075026 or cg20966754 were not particularly large, they were in the upper third of the distribution. Each of them showed moderate intrapair correlation rates (Spearman's rho = 0.809 and 0.762, respectively), indicating a role for unique environmental influences on their basis.

**Figure 2:**
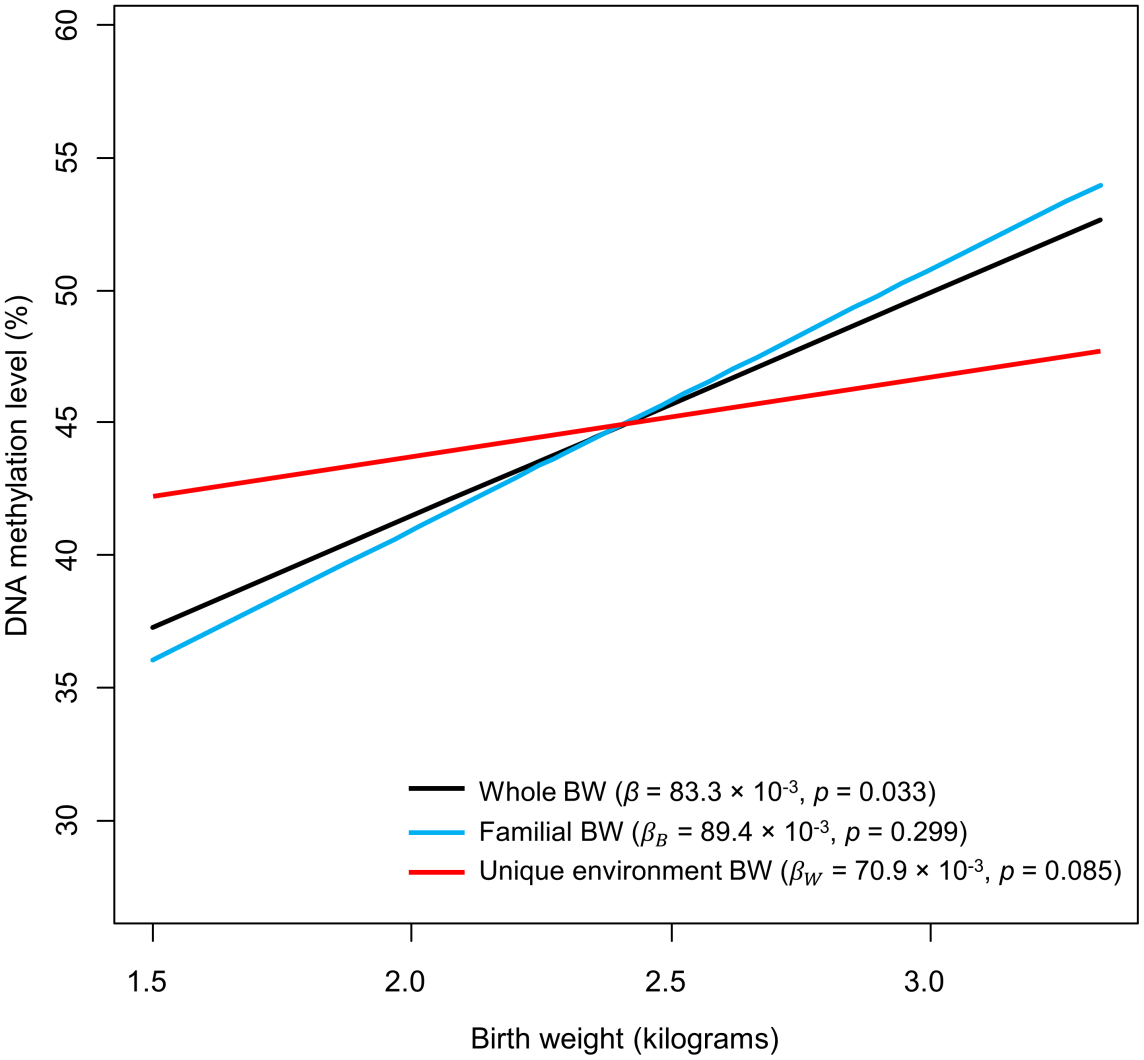
Representation of the association between birth weight and DNA methylation level of cg07075026 and cg20966754. The black line (“Whole BW”) was obtained from the first regression test (i.e., using raw BW from each of the 34 individuals), whereas blue and red lines (“Familial BW” and “Unique environment BW”) represent outcomes from the model evaluating familial and unique environmental factors.

**Figure 3:**
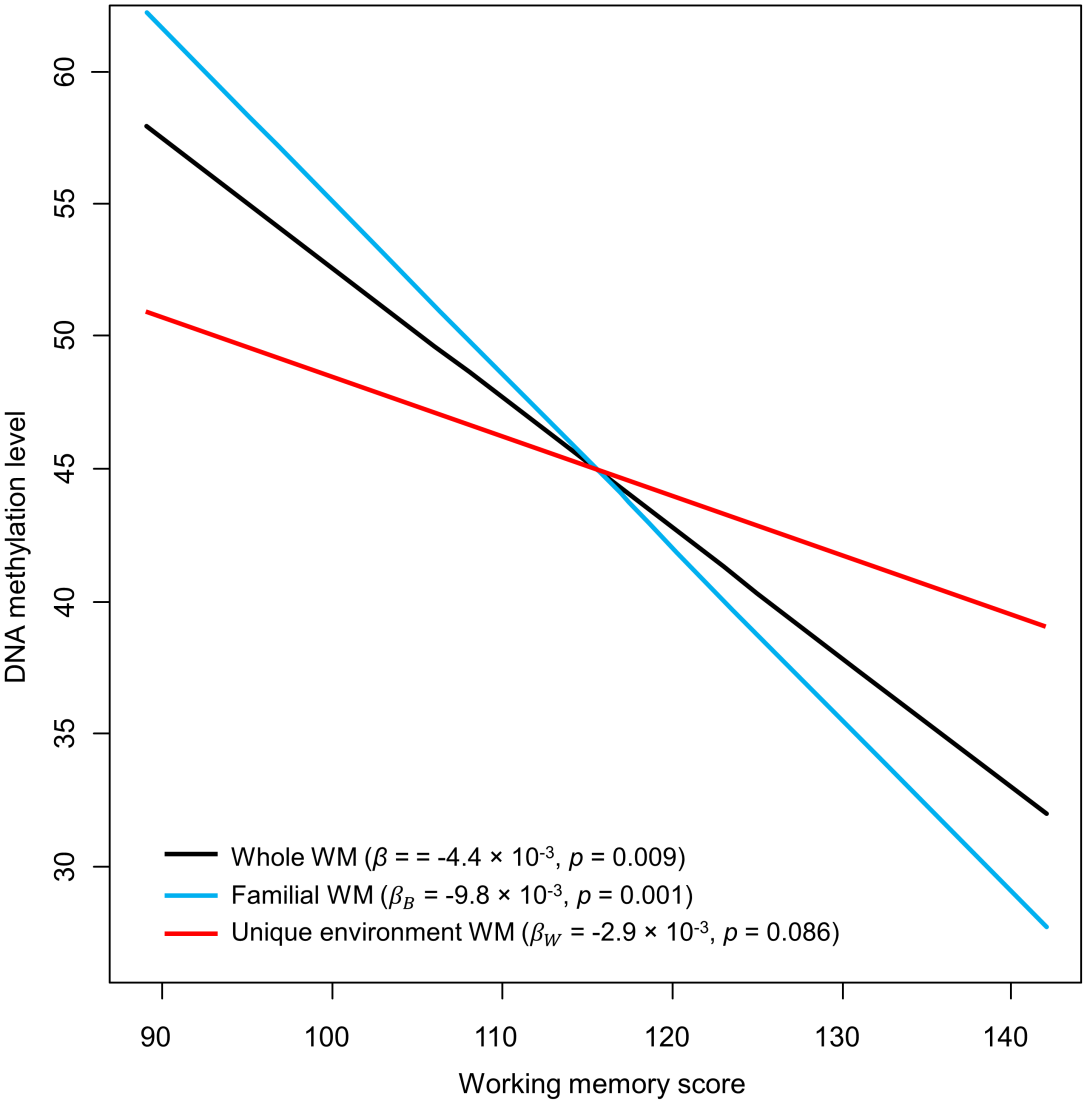
Representation of the association between working memory and DNA methylation level of cg07075026 and cg20966754. The black line (“Whole WM”) was obtained from the first regression test (i.e., using raw WM from each of the 34 individuals), whereas blue and red lines (“Familial WM” and “Unique environment WM”) represent outcomes from the model evaluating familial and unique environmental factors.

Associations were detected between *IGF2BP1* methylation fraction and both BW (*β* = 83.3×10^−3^, *p* = 0.033) and WM (*β* = −4.4×10^−3^, *p* = 0.009) (see Table 3, Figure 2 and Figure 3). Thus, in this model, each BW kilogram increase correlated with approximately 8.33% rise in methylation fraction, whereas a 10-point upsurge in WM performance score would be associated to a 4.4% methylation level reduction.

**Table 3:** Results of the linear regression testing the association between *IGF2BP1* DNA methylation levels and both BW and WM.

	*β*	SE	*t*	Pr(>|*t*|)
**Birth weight**	83.3×10^−3^	0.037	2.245	0.033
**Working memory**	−4.4×10^−3^	0.002	−2.775	0.009

Mean methylation percentage of cg07075026 and cg20966754 was used as outcome. Analyses were adjusted for gender, age, weeks of gestation and IQ, and accounted for correlated responses from twin pairs using a mixed effects model. BW was introduced in kilograms and WM in standard units. SE: Standard error.

Besides, analyses of familial and unique environmental influences indicated that the association between methylation and BW may be due to unique environmental influences on BW. Nonetheless, this result was statistically significant only at a trend level (*β_B_* = 89.4×10^−3^, *p* = 0.299; *β_W_* = 70.9×10^−3^, *p* = 0.085) (see Table 4 and Figure 2); hence, in this regression, every kilogram of intrapair advantage over the pair's mean BW value would be associated with a 7.09% increase in DNA methylation. The relationship between WM and *IGF2BP1* methylation was mainly due to shared genetic and environmental factors, although unique environmental influences also showed a trend towards significance (*β_B_* = −9.8×10^−3^, *p* = 0.001; *β_W_* = −2.9×10^−3^, *p* = 0.086) (see Table 4 and Figure 3): 10-point rises in the pair's mean WM score (i.e., WM's familial component) would account for an approximate reduction of 9.8% in methylation, whereas a 10-point advantage over a duo's average WM value would correlate with a 2.9% methylation level reduction.

**Table 4:** Results of the linear regression testing the association between *IGF2BP1* DNA methylation level and the familial and unique environmental factors of both BW and WM.

	*β*	SE	*t*	Pr(>|*t*|)
**Birth weight**				
Familial factors (*β_B_*)	89.4×10^−3^	0.084	1.061	0.299
Unique environment (*β_W_*)	70.9×10^−3^	0.039	1.794	0.085
**Working memory**				
Familial factors (*β_B_*)	9.8×10^−3^	0.003	−3.669	0.001
Unique environment (*β_W_*)	−2.9×10^−3^	0.002	−1.785	0.086

Mean methylation percentage of cg07075026 and cg20966754 was used as outcome. Analyses were adjusted for gender, age, weeks of gestation and IQ, and accounted for correlated responses from twin pairs using a mixed effects model. BW was introduced in kilograms and WM in standard units. SE: Standard error.

## Discussion

The current work suggests a putative link between both fetal growth and adult WM, and peripheral blood DNA methylation signatures at a region in the *IGF2BP1* gene, in agreement with previous literature [51], [52]. Besides, while the former reports separately detected associations between DNA methylation and either early development or WM, current results expand on the subject to indicate that, in the ongoing independent sample, relationships between *IGF2BP1* DNA methylation and either BW or WM phenotypes are not confounded by each other. In view of the current working hypothesis, results aid to speculate that *IGF2BP1* methylation levels may be determined by early environmental factors and that later compensatory brain mechanisms in healthy individuals could participate in raising cognitively normal profiles.

IGF2BP1 is a member of the highly conserved VICKZ (Vg1 RBP/Vera, IMP1-3, CRDBP, KOC, and ZBP1) family of RNA-binding proteins [53]. Remarkably, several functions within the central nervous system have previously been described for the IGF2BP1 and other members of VICKZ, suggesting their involvement in synaptic plasticity and hippocampal development. For instance, ZBP1 has been shown to interact with BDNF to regulate plasticity [54] and influence growth cone guidance [55], [56]. Besides, ZBP1 participates in prenatal hippocampal cell signaling and development and signaling [57], [58].

As regards potential functional consequences of the presently found epigenetic signature, it is worth mentioning that, while hypermethylation has typically been associated with gene silencing, recent evidence indicates this is not always true, and the inverse relationship has also been detected across several genomic regions [59]. Increased methylation of intragenic regions generally correlates with increased transcription [60], [61]. Hence, one could posit a direct correlation between methylation and gene expression at the locus discussed here in *IGF2BP1*. Speculation on the directions of regression slopes obtained here should be accordingly derived. First, lower BW could correlate with gene silencing and reduced protein activity; secondly, since WM consolidation takes place during childhood and later developmental windows, healthy individuals (such as those in this sample) with reduced *IGF2BP1* transcription may have improved their WM performance to counteract potentially harmful effects of growth impairments. Further conjectures are elaborated below.

Concerning human fetal growth, it is worth noticing that a recent manuscript found an association between human fetal leukocyte DNA methylation of the *IGF2BP1* and gestational age [51], thus indicating that methylation of this gene could be a marker of developmental impairment. However, the 6 *IGF2BP1*‘s CpG sites these authors found associated with gestational age showed neither inter-individual nor intrapair variability in this sample (see Fig. 1).

Furthermore, it has been suggested that some DNA methylation marks in adults may correlate with prenatal trajectories [10]. In view of it, the fact that current multivariate analyses detected a negative correlation between WM and methylation may lead to hypothesize that individuals who suffered early insults –which could have established long-lasting epigenetic signatures–, might raise some cognitive skills in order to attenuate/counteract the impact of such developmental injuries. In fact, a recent compensatory scheme of the neurodevelopmental underpinnings of schizophrenia suggests that adaptation reactions may arise in individuals who suffer early impairments, and thus disease status would be a consequence of a failure of the compensatory response [62]. Hence, as this study considered adults from the general population, one may speculate that the early impact of low BW on *IGF2BP1* methylation status may later be lessened by WM performance improvements.

In a second set of analyses, decomposing BW and WM into both familial and unique environmental components allowed detecting that the BW-*IGF2BP1* methylation may be due to unique environmental influences. Other studies have described a number of maternal, fetal and placental sources of twin BW discordance (i.e., specific intrauterine conditions which could account for the aforesaid “unique environment”) [63]. Notably, since both genes and environment shape the human neonatal epigenome [64], it is worth mentioning that previous reports have indicated intrapair DNA methylation differences in between heaviest and lightest newborn twins [65]. Nonetheless, other studies of DNA methylation in adult twins who were discordant for BW have failed to detect this association, probably due to the methodological limitations of using peripheral DNA samples [66], among other factors such as between-study sample heterogeneity.

Although less studied, there is some evidence indicating that adult WM performance could correlate with peripheral blood DNA methylation levels [52]. While in a different locus, the present twin study suggests the presence of a WM-methylation link, and also points that it may be driven by both familial and unique environmental factors. As adult WM is influenced by genes and environment [67], the same may be proposed for its relationship with DNA methylation, even though further research is needed to disentangle this potential relationship. Furthermore, since hippocampal synaptic plasticity influences WM [68], it is not surprising that Mukhopadhyay et al. [69] described how intrauterine insults may alter *IGF2BP1* gene expression in the developing hippocampus and cause long-term cognitive damage through functional compromise of hippocampal neurons.

Additionally, it is worth noting that both BW and WM were studied in relation to epigenetic changes in molecular pathways involving the IGF2 family. Thus, the direction of associations found here may be limited to the locus studied. Moreover, as intrapair differences in methylation percentage across all 248 CpG sites initially considered were small (median difference at each CpG <5.5%), overall methylation profiles must have been highly influenced by genetic factors. Besides, while genome-wide DNA methylation profiles may be influenced by single nucleotide polymorphisms (SNPs), data from dbSNP 138 [38], [70] indicates there are no validated common SNPs in the genomic loci of these CpG sites for European descent populations.

A final limitation of this work should be noted apropos the relationship between DNA methylation in peripheral blood and brain regions. Although large epigenetic differences between some tissues have been documented in previous studies [71], [72], some evidence from animal research suggests correlation between DNA methylation patterns across peripheral lymphocytes and a number of brain regions, presumably reflecting early environmental exposures [73]–[75]. Accordingly, the growing amount of publications in the literature showing significant DNA methylation alterations in peripheral cells of individuals with mental health conditions [76] suggests that this epigenetic mark, as measured in blood, could be suitable for research of complex brain-related phenotypes. Also, other authors have summarized published studies of psychiatric disorders, to suggest a high correlation between blood and brain methylation signatures [77]. Nevertheless, while a blood/brain DNA methylation correlation may exist for the genomic region studied here, the argument is still speculative and future research should correspondingly address the issue. As long as this study is exploratory, results must be taken with caution. Replication of the findings is needed in larger independent samples.
